# Phenotypic continuum of *NFU1* ‐related disorders

**DOI:** 10.1002/acn3.51679

**Published:** 2022-10-18

**Authors:** Rauan Kaiyrzhanov, Maha S. Zaki, Tracy Lau, Sambuddha Sen, Reza Azizimalamiri, Mina Zamani, Gözde Yeşil Sayin, Taru Hilander, Stephanie Efthymiou, Viorica Chelban, Ruth Brown, Kyle Thompson, Maria Irene Scarano, Jaya Ganesh, Kairgali Koneev, Ismail Musab Gülaçar, Richard Person, Dinara Sadykova, Yerdan Maidyrov, Tahereh Seifi, Aizhan Zadagali, Geneviève Bernard, Katrina Allis, Houda Zghal Elloumi, Amanda Lindy, Ehsan Taghiabadi, Sumit Verma, Rachel Logan, Brian Kirmse, Renkui Bai, Shaimaa M. Khalaf, Mohamed S. Abdel‐Hamid, Alireza Sedaghat, Gholamreza Shariati, Mahmoud Issa, Jawaher Zeighami, Hasnaa M. Elbendary, Garry Brown, Robert W. Taylor, Hamid Galehdari, Joseph J. Gleeson, Christopher J. Carroll, James A. Cowan, Andres Moreno‐De‐Luca, Henry Houlden, Reza Maroofian

**Affiliations:** ^1^ Department of Neuromuscular Disorders UCL Queen Square Institute of Neurology London WC1N 3BG UK; ^2^ Human Genetics and Genome Research Division, Clinical Genetics Department National Research Centre Cairo Egypt; ^3^ Department of Chemistry and Biochemistry The Ohio State University 100 West 18th Avenue Columbus Ohio 43210 USA; ^4^ Department of Paediatric Neurology, Golestan, Medical, Educational, and Research Center Ahvaz Jundishapur University of Medical Sciences Ahvaz Iran; ^5^ Department of Biology, Faculty of Science Shahid Chamran University of Ahvaz Ahvaz Iran; ^6^ Department of Medical Genetics, Istanbul Faculty of Medicine Istanbul University Istanbul 34098 Turkey; ^7^ Genetics Section, Molecular and Clinical Sciences St George's, University of London London UK; ^8^ Oxford Medical Genetics Laboratories The Churchill Hospital Oxford OX3 7LJ UK; ^9^ Wellcome Centre for Mitochondrial Research, Translational and Clinical Research Institute Faculty of Medical Sciences Newcastle University Newcastle upon Tyne NE2 4HH UK; ^10^ Division of Genetics, Cooper Health System Children's Regional Hospital Sheridan Pavilion Camden New Jersy 08103 USA; ^11^ Department of Genetics and Genomic Sciences Icahn School of Medicine at Mount Sinai New York New York USA; ^12^ Department of Neurology and Neurosurgery Asfendiyarov Kazakh National Medical University Almaty 050000 Kazakhstan; ^13^ Department of Genetics Institute of Graduate Studies in Health Sciences, Istanbul University Istanbul 34098 Turkey; ^14^ GeneDx Gaithersburg Maryland 20877 USA; ^15^ Astana Medical University Nur‐Sultan Kazakhstan; ^16^ L.N. Gumilyov Eurasian National University Nur‐Sultan Kazakhstan; ^17^ Departments of Neurology and Neurosurgery, Pediatrics and Human Genetics McGill University Montreal Canada; ^18^ Division of Medical Genetics, Department Specialized Medicine McGill University Health Centre Montreal Canada; ^19^ Child Health and Human Development Program Research Institute of the McGill University Health Centre Montreal Canada; ^20^ Skin and Stem Cell Research Center, Tehran University of Medical Sciences Tehran Iran; ^21^ Department of Neurology Emory University School of Medicine Georgia Atlanta USA; ^22^ Division of Neurosciences Children's Healthcare of Atlanta Atlanta Georgia USA; ^23^ Division of Genetics University of Mississippi Medical Center Jackson Mississippi USA; ^24^ Pediatrics Department Assiut University Assiut Egypt; ^25^ Medical Molecular Genetics Department Human Genetics and Genome Research Institute, National Research Centre Cairo Egypt; ^26^ Health Research Institute, Diabetes Research Center Ahvaz Jundishapur University of Medical Sciences Ahvaz Iran; ^27^ Department of Medical Genetics, Faculty of Medicine Ahvaz Jundishapur University of Medical Sciences Ahvaz Iran; ^28^ Narges Medical Genetics and Prenatal Diagnosis Laboratory East Mihan Ave., Kianpars Ahvaz Iran; ^29^ NHS Highly Specialised Service for Rare Mitochondrial Disorders Newcastle upon Tyne Hospitals NHS Foundation Trust Newcastle upon Tyne NE1 4LP UK; ^30^ Department of Neurosciences University of California, San Diego La Jolla California 92093 USA; ^31^ Rady Children's Institute for Genomic Medicine San Diego California 92025 USA; ^32^ Department of Radiology Autism & Developmental Medicine Institute, Genomic Medicine Institute Geisinger Danville Pennsylvania 17822 USA

## Abstract

Bi‐allelic variants in Iron–Sulfur Cluster Scaffold (*NFU1*) have previously been associated with multiple mitochondrial dysfunctions syndrome 1 (MMDS1) characterized by early‐onset rapidly fatal leukoencephalopathy. We report 19 affected individuals from 10 independent families with ultra‐rare bi‐allelic *NFU1* missense variants associated with a spectrum of early‐onset pure to complex hereditary spastic paraplegia (HSP) phenotype with a longer survival (16/19) on one end and neurodevelopmental delay with severe hypotonia (3/19) on the other. Reversible or irreversible neurological decompensation after a febrile illness was common in the cohort, and there were invariable white matter abnormalities on neuroimaging. The study suggests that MMDS1 and HSP could be the two ends of the *NFU1*‐related phenotypic continuum.

## Introduction

Iron–sulfur [Fe‐S] clusters are important cofactors that play a role in various cellular functions, including electron transfer along the respiratory chain, citric acid cycle, heme biosynthesis, DNA replication and repair, as well as iron homeostasis.^1^, ^2^, ^3^ Iron–Sulfur Cluster Scaffold (*NFU1*) (MIM: 608100) is an [Fe‐S] cluster biosynthesis factor involved in the last steps of maturation and transfer of [4Fe‐4S] clusters into target proteins, including lipoic acid synthetase, mitochondrial aconitase and some subunits of respiratory chain complexes I and II.^4^, ^5^ Bi‐allelic variants in *NFU1* have previously been associated with multiple mitochondrial dysfunctions syndrome 1 (MMDS1)^6^ characterized by early‐onset leukoencephalopathy leading to a fatal outcome, typically before the age of 15 months.^6^

Recently, bi‐allelic variants in *NFU1* have been reported in two individuals with a milder phenotype, presenting with slowly progressive spastic paraplegia with a relapsing–remitting course, long survival, and intact cognition or mild intellectual disability (ID).^7^, ^8^ Here, we report 19 affected individuals from 10 independent families with ultra‐rare bi‐allelic *NFU1* missense variants associated with a phenotype ranging from early‐onset pure to complex hereditary spastic paraplegia (HSP) characterized by a longer survival (16/19) and neurodevelopmental delay (NDD) with severe hypotonia (3/19).

## Methods

### Participants and clinical investigations

Exome sequencing (ES) and genome sequencing (GS), data sharing between international genetic centers, and the GeneMatcher platform^9^ were employed to identify the 10 families reported here. Clinical data were collected via a uniform proforma. Parents/legal guardians of all affected individuals consented to the publication of genetic and clinical information. The study was approved by The Research Ethics Committee Institute of Neurology University College London (IoN UCL) (07/Q0512/26) and the local Ethics Committees of each participating center.

### Exome sequencing and genome sequencing

Research/diagnostic solo or trio‐ES/GS, variant filtering, and variant confirmation by Sanger sequencing and segregation analysis were performed on genomic DNA extracted from blood in different genetic laboratories following the methods previously described (Table 1). Family 10 had a mitochondrial panel test performed for the proband and targeted genotyping for familial variants for the sibling (further details in Table 1 and Supplementary Table S1).

**Table 1:** Clinical features of the present cohort and description of *NFU1* variants.

	F1 (F1‐II:1,F1‐II:2)	F2 (F2‐II:1)	F3 (F3‐II:1, F3‐II:2)	F4 (F4‐II:4, F4‐II:5,‐F4‐II:6)	F5 (F5‐II:1, F5‐II:2)	F6 (F6‐II:2, F6‐II)	F7 (F7‐II:1, F7‐II:2)	F8 (F8‐II:5, F8‐II)	F9 (F9‐II:1)	F10 (F10‐II:1, F10‐II:2)	Previously published reports with HSP phenotype	
Reported in	This study	This study	This study	This study	This study	This study	This study	This study	This study	This study	Tonduti et al., 2015	Uzunhan et al., 2020
Ethnic origin	Iranian‐Arab	American	Egyptian	Egyptian	Egyptian	Egyptian	Kazakh	Turkish	American	European‐American	Not reported	Not reported
Consanguinity	Yes	No	Yes	Yes	Yes	Yes	No	Yes	No	No	No	Yes
Genetic investigation	ES	ES	ES	ES	ES	ES	ES	GS	ES	NGS Panel Mitochondrial Disorders	Not reported	ES
WES/WGS and variant filtering method	Makrythanasis et al., 2018^10^	Guillen Sacoto et al., 2020^11^	Makrythanasis et al., 2018^10^				Makrythanasis et al., 2018^10^	Sefid et al., 2017^12^; Cirulli et al., 2010^13^	Guillen Sacoto et al., 2020^11^	Lindy et al., 2018^14^	Tonduti et al., 2015^7^	Uzunhan et al., 2020^8^
Variant at cDNA level*	c.721G>C	c.298G>C; c.301A>G	c.362T>C				c.295C>G	c.263T>C	c.548C>G; c.629G>T	c.629G>T; c.398T>C	c.146delC; c.565G>A	c.565G>A
Variant at protein level*	p.(Val241Leu)	p.(Ala100Pro); p.(Arg101Gly)	p.(Val121Ala)				p.(Leu99Val)	p.(Phe88Ser)	p.(Pro183Arg); p.(Cys210Phe)	p.(Cys210Phe); p.(Leu133Pro)	p.(Pro49LeufsTer8); p.(Gly189Arg)	p.(Gly189Arg)
Sex	2F	1M	1M, 1F	3F	1M, 1F	2M	2M	2M	1M	1M, 1F	1M	1F
Age at last examination	10y and 17 y	25 y	9 y and 12 y	2 y and 6 y	4,5 m and 6 m	3 y and 2.2 y	10 y and 6 y	7.5 and 6.5 y	12 y	1 y and 2 y	30 y	3.5 y
Age of onset	6–9 m and 9–10 m	1 y	1 y (2)	0.9 y and 1,2 y	1,1 y and 0.5 y	1 y and 0.9 m	3–4 m (2)	18 m (2)	2 y	6 m and 3 m	18 m	23 m
Disease duration	9 y and16 y	24 y	8 y and 11 y	10 m (1), 6 m (1), and 5 y (1)	Died at 6 m (1) and 3y (1)	2 y and 1.6 y	10 y and 6 y	6 y and 5 y	10 y	Died at 13 m and 25 m	28.5 y	21 m
Family history	Yes	No	Yes (2)	Yes (3)	Yes (2)	Yes (2)	Yes (2)	Yes (2)	No	Yes (1), No (1)	No	Yes
Mortality	Alive (2)	Alive	Alive (2)	Died at 33 m (1) and 30 m (1), alive (1)	Died (1) and alive (1)	Deceased (1)	Alive (2)	Alive (2)	Alive	Died after febrile illness (2)	Alive	Alive
Onset with delayed walking/progressive spastic paraplegia	Yes (2)	Yes	Yes (2)	Yes (3)	Yes (1)	Yes (2)	Yes (2)	Yes (2)	Yes	No (2)	Yes (2)	Yes
Mild ID/Cogitative impairment	No (2)	No	Yes (2)	Yes (1)	No (2)	No (2)	No (2)	No (2)	No	Yes (2)	Yes	Yes
Lower limb spasticity	Yes (2)	Yes	Yes (2)	Yes (3)	Yes (1)	Yes (2)	Yes (2)	Yes (2)	Yes	Yes (1)	Yes	Yes
Deterioration with loss of milestones with intercurrent viral/febrile illness	Yes	Yes	No (2)	Yes (2), No (1)	Yes (2)	Yes (2)	No (2)	Yes (2)	Yes	Yes (2)	Yes	Yes
Recovery of loss of milestones/previous status after recovery from illness	Yes (1), NA (1)	Yes	NA (2)	No (2), NA (1)	No (1) and Yes (1)	Yes (2)	NAP	No (1), NA (1)	Yes	No (2)	Yes	Yes
Mild WMHs in T2WI	Yes (2)	No**	No (2) **	Yes (2)	Yes (1)	Yes (1), NA (1)	Yes (1), NA (1)	Yes (1) ***, NA (1)	No**	Yes (2)	Yes	Yes
Mild T2 hyperintensity in BG	Yes (2)	No**	No (2) **	Yes (2)	Yes (1), No (1)	Yes (1)	Yes (1), NA (1)	Yes (1) ***, NA (1)	No**	Yes (2)	No	No
CC abnormalities	Yes (2)	No**	No (2) **	Yes (2)	Yes (1), No (1)	Yes (1)	Yes (1), NA (1)	No (1), NA (1)	No**	Yes (2)	Yes	Yes
Areas of cystic degeneration/leukomalacia	No (2)	No**	No (2) **	No (2)	No (2)	No	No (1), NA (1)	No (1), NA (1)	No**	Yes (1), No (1)	No	Yes

ES, exome sequencing; GS, genome sequencing; WES, Whole exome sequencing; WGS, Whole genome sequencing; NGS, next generation sequencing; F, female; M, male; y, year(s); m, month(s); NAP (no applicable); T2WI, T2 weighted images; BG, basal ganglia; CC, corpus callosum.

^*^ Based on the transcripts NM_001002755.4 & NP_001002755.1 respectively.

^**^ Reported as normal. Study not independently reviewed.

^***^ Questionable, please refer to Supplementary Table S1.

Allele frequencies of the variants, in silico predictions, and ACMG classifications are provided in Supplementary Table S1.

### SDS‐PAGE and western blot analysis

Human fibroblasts from an affected individual (F1‐II:1) were obtained and lysed and samples were subjected to SDS‐PAGE and western blotting as described previously^15^ and a list of primary antibodies is provided (Supplementary Methods).

### Protein modeling and characterization, Fe‐S cluster transfer monitoring, and enzymatic assays

Protein characterization by variable temperature circular dichroism (CD) and analytical ultracentrifugation, Fe‐S cluster transfer monitored by circular dichroism, pyruvate dehydrogenase and α‐ketoglutarate dehydrogenase activity assay studies were performed (Supplementary Methods).

## Results

### Clinical phenotype

**Figure 1:**
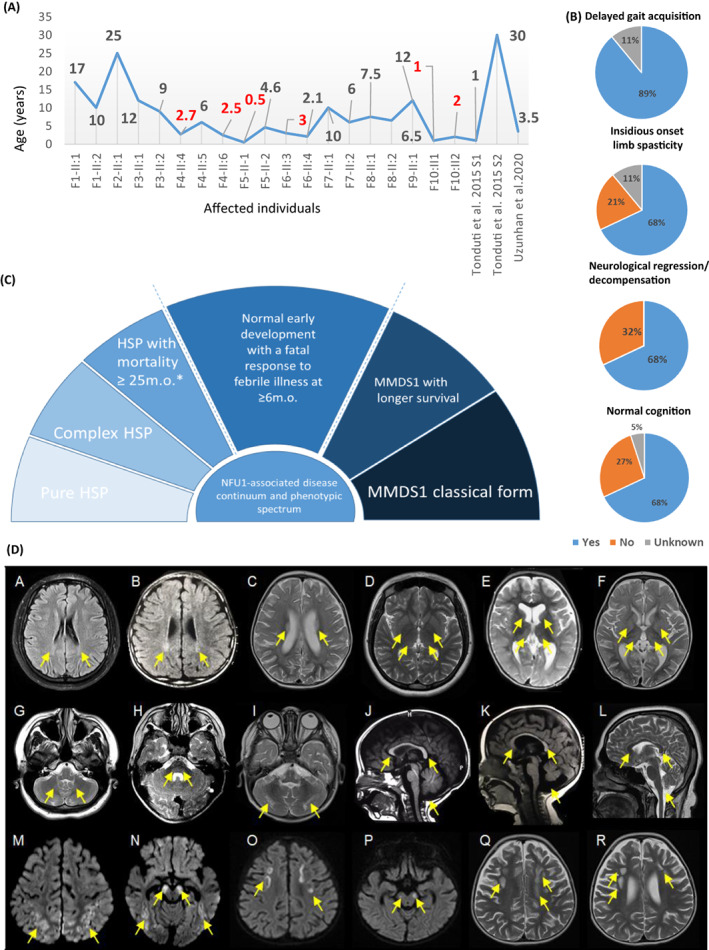
Clinical features of the individuals reported in this study and *NFU1*‐associated phenotypic continuum. (A) Ages of the affected individuals at the study recruitment. (B) Clinical features of the present cohort. (C) *NFU1*‐associated phenotypic continuum. HSP, hereditary spastic paraplegia. (D) Representative brain MRI features of the present cohort. Individual F1‐II:2 (A, D, G, L), individual F4‐II:5 (B), individual F10‐II:1 (C, F, I, M, N), individual F5‐II:2 (E, K), individual F7‐II:1 (H), individual F4‐II:6 (J), and individual F10‐II:2 (O, P, Q, R). T2/FLAIR hyperintense signal involving the bilateral posterior centrum semiovale (Q), corona radiata, and periatrial regions (A‐C). T2 hyperintense signal involving the bilateral thalami and basal ganglia (D‐F), pons, and cerebellum (G‐I). Hypoplastic corpus callosum and mega cisterna magna (J‐L). Bilateral cerebral white matter volume loss (A‐F, Q, R). Areas of restricted diffusion involving the bilateral subcortical white matter and cerebral peduncles (M‐P). Areas of cystic degeneration/leukomalacia in the white matter of the bilateral frontal lobes (Q, R). Vermian hypoplasia (K).

The cohort comprises 19 affected individuals from 10 independent families. There were 8 females and 11 males, 13 of whom are currently alive with a mean age of 9.8 ± 5.7 years (range 2.1–25) at the study recruitment (Fig. 1A). Six affected individuals succumbed to their rapidly progressive disease course triggered by a febrile illness between the ages of 6–36 months. Detailed clinical information is provided in Supplementary Table S1 and Supplemental Case Reports.

Prenatal features and neonatal periods were unremarkable in all subjects. No delay in early developmental milestones including head control, crawling, sitting, and speech was reported in 14/19 individuals. Cognitive function was impaired in 5/18 individuals. Gait acquisition was delayed due to either the insidious onset of limb spasticity in 13/19 individuals or spasticity precipitated by a deterioration in the context of a febrile illness in 4/19 individuals (Fig. 1B). The lower limb spasticity was detected at a mean age of 12 ± 6 months in the cohort. Spasticity was progressive leading to contractures in 13/19 persons and necessitating Achilles' tendon repair surgery in 4/19 affected individuals.

Remarkably, febrile illness leading to metabolic decompensation played a significant role in the disease course of 13/19 individuals. Thus, in F1‐II:1 it led to febrile seizures followed by the onset of lower limb spasticity and reversible cognitive and motor regression, and truncal hypotonia. Similarly, F5‐I:1, F6‐II:3, and F8‐II:5 developed reversible truncal hypotonia and/or progressive lower limb spasticity following several episodes of febrile illness. Intermittent and reversible gait deterioration associated with episodes of febrile illness was reported in F2‐II:1and F9‐II:1, along the course of their disease, which was described as waxing and waning. While the episodes of febrile illness had not been fatal for these persons, the other six affected individuals had regression and developed severe muscular hypotonia leading to early mortality after a febrile illness.

The mean disease duration at the most recent examination for the living individuals was 8.8 ± 5.7 years (range 1.5–24). Neuromuscular examination revealed lower limb spasticity in all persons with increased deep tendon reflexes, upgoing plantar reflexes, and mild symmetrical muscle wasting. Muscle tone from the upper limbs was uniformly intact. While two individuals were wheelchair‐bound, eight could ambulate using crouches or rollator walker, and three could walk independently with spastic gait. Ataxic gait was reported in 2/12 individuals, which was intermittent in one person (F2‐II:1). The oldest individual in the cohort was aged 25 years and had a normal cognitive function and could perform most activities of daily living despite his slowly progressive lower limb spasticity. Seven persons had results available of metabolic screening, which were unremarkable in all.

Brain MRI DICOM files were available for six individuals (F1‐II:1, F1‐II:2, F7‐II:1, F8‐II:5, F10‐II:1, and F10‐II:2), low‐resolution photos of brain MRI studies were available for four individuals (F4‐II:5, F4‐II:6, F5‐II:2, and F6‐II:2), and photos of a head CT in one person (F5‐II:1). All available neuroimaging studies were independently reviewed by a board‐certified neuroradiologist. Four additional individuals had neuroimaging studies that were reported as normal but not independently reviewed (F2‐II:1, F3‐II:1, F3‐II:2, and F9‐II:1). Shared neuroimaging findings included T2 hyperintense signal involving the bilateral posterior centrum semiovale, corona radiata, and periatrial regions (9/9, 100%); T2 hyperintense signal involving the bilateral thalami (9/9, 100%), basal ganglia (7/9, 78%), and pons and cerebellum (7/8, 88%), hypoplastic corpus callosum (9/10, 90%), bilateral cerebral white matter volume loss (8/11, 73%), mega cisterna magna (9/11, 82%), mild prominence of the lateral ventricles and frontoparietal sulci (7/11, 64%), and scattered areas of restricted diffusion which may represent active demyelination, metabolic encephalopathy, or acute/subacute ischemia (3/4, 75%). A single individual (1/11, 9%) had areas of cystic degeneration/leukomalacia in the white matter of the bilateral frontal lobes and another (1/11, 9%) had vermian hypoplasia. Figure 1D shows shared neuroimaging findings and the Supplemental information has case‐based descriptions of the brain MRI/CT interpretations.

### Genetic analysis reveals ultra‐rare bi‐allelic variants in *NFU1*

**Figure 2:**
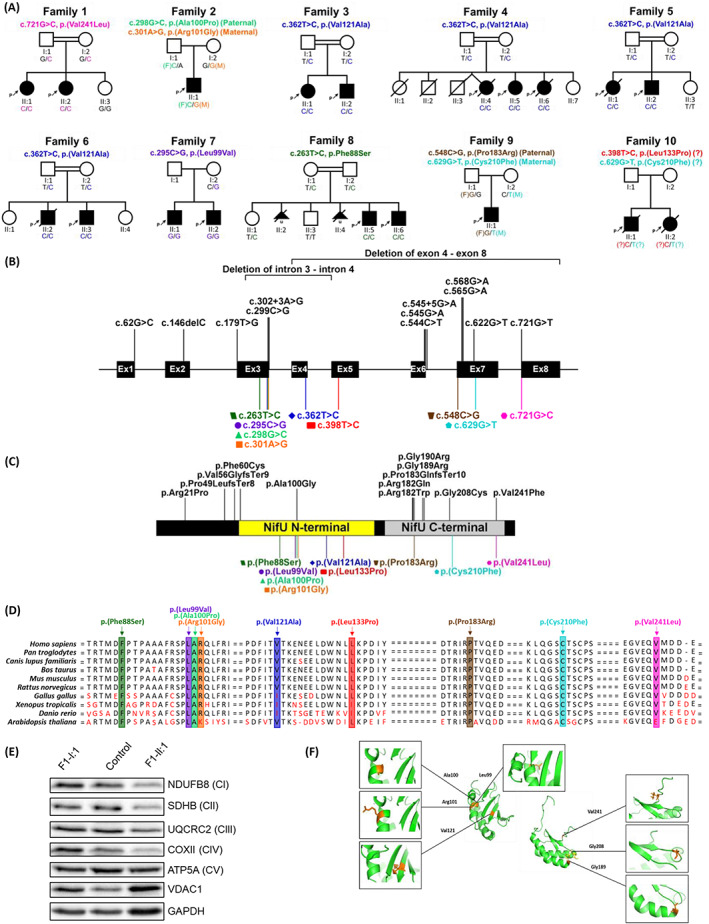
Overview of the genetic and biochemical characteristics of *NFU1* variants. (A) Pedigrees and segregation results of the ten families included in this study. (B) *NFU1* gene structure with the localization of all previously known mutations (black, above) and mutations reported in this cohort (colored, below). The corresponding amino acid changes is shown in (C) with known protein domains in NFU1 (NifU N‐terminal domain, residue 59 to 155, and NifU C‐terminal domain, residue 162 to 247) depicted in the illustration. The variant, p.(Val241Leu), was the only mutation discovered in this study to have been located outside of the NFU1 protein domains. Regions are not drawn to scales and both illustrations were created from the program.^16^ (D) Conservation of the nine mutations reported in the present cohort across 10 species. (E) Western blot analysis of structural subunits from each OXPHOS complex (CI [NDUFB8], CII [SDHB], CIII [UQCRC2], CIV [COXII], and CV [ATP5A]) in fibroblasts from F1‐II:1 (affected individual), F1‐I:1 (mother) and a pediatric control. GAPDH and VDAC1 were used as cell and mitochondrial loading controls, respectively. (F) Protein model of the NifU N‐terminal domain and NifU C‐terminal domain of the NFU1 structure. Positions of amino acids affected by *NFU1* missense variants are indicated in orange.

A total of 9 different *NFU1* missense variants were identified in this study (Figs. 2A to C). Exome sequencing carried out in the proband F1‐II:1 revealed a homozygous likely pathogenic *NFU1* variant: NM_001002755.4:c.721G>C, p.(Val241Leu). In the third affected individual F2‐II:1, trio‐ES identified compound heterozygous *NFU1* variants: a variant of uncertain significance (VUS), c.298G>C, p.(Ala100Pro) inherited from the father and a VUS, c.301A>G, p.(Arg101Gly) inherited from the mother.

Exome sequencing of four unrelated Egyptian families, F3 to F6, led to the identification of the same pathogenic homozygous *NFU1* variant: c.362T>C, p.(Val121Ala). A homozygous VUS, c.295C>G, p.(Leu99Val) was also identified by ES of the proband of family 7. In family 8, a homozygous VUS, c.263T>C, p.(Phe88Ser) was identified by ES of the proband. Trio‐ES in family 9 found compound heterozygous *NFU1* variants: a maternally inherited likely pathogenic variant, c.629G>T, p.(Cys210Phe) and a paternally inherited VUS, c.548C>G, p.(Pro183Arg). In family 10, the combination of a mitochondrial gene panel and Sanger sequencing revealed potential compound heterozygous *NFU1* variants: a VUS, c.629G>T, p.(Cys210Phe) and a VUS, c.398T>C, p.(Leu133Pro). Apart from, p.(Leu99Val), all the homozygous variants are residing within sizeable regions of homozygosity. All the variants are either absent or observed in extremely low allele frequencies in numerous population variant frequency databases (~2 mln alleles). They affect highly conserved residues across different species (Fig. 2D) and segregated with the disease phenotype in all the families (Fig. 2A). No other relevant variants associated with neurological or neurodevelopmental disorders were identified in the currently known monogenic disease‐causing genes in the ES data.

The characteristics of the variants are further summarized in Table 1 and Supplementary Table S1.

### Steady‐state levels of OXPHOS complexes I, II, III, and IV are decreased in homozygous p.(Val241Leu) fibroblasts

Western blot analysis on fibroblast samples showed that subunits of each complex of the electron transport chain (CI‐CIV) were decreased in the affected individual F1‐II:1 compared to the heterozygous mother (F1‐I:1) and a pediatric control, whereas levels of complex V subunit ATP5A remained relatively unchanged (Fig. 2E).

### Functional study of the p.(Val241Leu) variant

Protein modeling revealed all nine variants were located in the NFU1 protein domains. Six variants, p.(Phe88Ser), p.(Leu99Val), p.(Ala100Pro), p.(Arg101Gly), p.(Val121Ala), and p.(Leu133Pro), were found in the NifU N‐terminal domain while the remaining three, p.(Pro183Arg), p.(Cys210Phe), and p.(Val241Leu), lie in the NifU C‐terminal [Fe‐S] cluster binding domain (Figs. 2C,F, Supplementary Results). The p.(Val241Leu) variant was further investigated via *in vitro* [Fe‐S] cluster reconstitution experiments, however, the results showed no significant change in the protein's secondary structure (Supplementary Fig. S1C, Supplementary Results).

Additional discussion of the structural and functional biochemistry of other variants is provided in the Supplementary Material. As patient fibroblasts were available only for the p.(Val241Leu) variant, the impact of the variant on the enzymatic activities of PDH and KGDHC was measured only for this variant. Enzymatic activity assays in fibroblasts derived from F1‐II:1, homozygous for p.(Val241Leu), showed decreased activity for PDH (0.50 nmol/mg protein/min; normal range 0.6–0.9 nmol/mg protein/min), while the mean activity in the heterozygous mother was normal (0.82 nmol/mg protein/min; normal range 0.6–0.9 nmol/mg protein/min). The KGDHC activity was not impaired (Supplementary Fig. S2, Supplementary Results).

## Discussion

The bioenergetic function of the mitochondrion is dependent on Fe‐S cluster‐containing proteins. Three distinctly organized biosynthetic pathways are involved in the maturation of Fe‐S clusters in mammals with the last step involving *NUBPL, NFU1, BOLA3, IBA57, ISCA2*, and *ISCA1*. Bi‐allelic pathogenic variants in five of them, starting from *NFU1* in the above‐mentioned list, are currently associated with MMDS types 1 to 5 respectively, and typically present with severe and fatal early onset encephalopathy with multiple biochemical abnormalities.^6^ An atypical presentation of MMDS has been reported in several individuals, mainly encompassing a slightly milder disease course with a longer survival^17^, ^18^, ^19^ for *NFU1* and *ISCA2*, or a complex HSP phenotype for *NFU1* and *IBA57*.^7^, ^8^, ^20^ Our report expands the number of individuals with bi‐allelic *NFU1* variants presenting with complex HSP and also describes previously unreported pure HSP phenotype, thereby suggesting that MMDS1 and HSP could be the two ends of the *NFU1*‐associated phenotypic continuum.

Pyramidal symptoms in the form of spastic tetraparesis have frequently been reported in individuals with typical MMDS1 presentation, highlighting the role of mitochondria and axonal transport in the corticospinal tract function.^6^ Currently, due to a large overlap between HSP and other inherited neurological disorders, at least 28/81 genetic forms of HSP are assigned alternative phenotypes on the Online Mendelian Inheritance in Man (OMIM) database, resulting in a diagnostic challenge.^21^ Therefore, delineating the full phenotypic spectrum of disease‐associated genes and accurate clinical classification are important for diagnostic rates.

Another remarkable aspect of the present cohort is the presence of neurological decompensation after a febrile illness. Interestingly, three distinct responses to episodes of febrile illness were observed in our cohort: (1) fatal outcome after the first or consecutive episode in six individuals; (2) onset of spasticity with variably reversible motor and cognitive regression in three individuals; and (3) intermittent and fully reversible gait deterioration in four other individuals. The episodes of neurological deterioration, most likely caused by acute illness‐induced metabolic decompensation, is common in mitochondrial diseases^22^ but have rarely been reported in the mitochondrial causes of HSP.^23^, ^24^

A remarkable inter‐and intrafamilial phenotypic variability was observed in the present cohort and the same amino acid substitutions of the NFU1 protein have been observed in both MMDS1 and HSP cases (Supplementary discussion for details). The relatively milder course and phenotypic variability of the most commonly reported MMDS1‐associated *NFU1* variant, c.565G>A, p.(Gly189Arg), was further supported in reports by Uzunhan et al. (2020) and Tonduti et al. (2015),^7^, ^8^ who described individuals with the same *NFU1* variant and presenting with HSP, with one individual reaching the age of 30 years. Defects in *NFU1* seem to present with a range of phenotype severities suggesting the clinical spectrum of *NFU1*‐associated disease (Fig. 1C). The constellation of shared neuroimaging findings noted in individuals for whom images were available is similar to the findings reported by Tonduti et al. (2015),^7^ including white matter abnormalities of the periventricular regions and thinning of the corpus callosum. Similarly, neuroimaging features present in our cohort overlap with some of those reported by Uzunhan et al. (2020).^8^ These include white matter hyperintensity, which was present in most of the individuals in the present series, cystic degeneration and cavitation in the frontal regions, which was noted in individual F10‐II:2, and areas of restricted diffusion involving the bilateral subcortical and periventricular white matter, noted in three out of four individuals with available diffusion‐weighted imaging.

Apart from the possible epigenetic factors,^7^, ^8^ we suspect that the variants observed in the present study might be causing milder defects for the NFU1 structure and function compared to the MMDS1‐linked variants. The different variants might affect, for example, partner protein binding in a distinct way leading to high phenotypic variability connected to *NFU1* variants (Supplementary Discussion for further details). The residual PDH activity and the normal KGDHC activity in F1‐II:1 might be consistent with a less severe form of the disease with prolonged survival. The decreased OXPHOS protein levels and the only slightly affected PDH and normal KGDHC activity results in F1‐II:1 fibroblasts might be explained by the changed affinity of the mutated NFU1 for the different partner proteins (Supplementary Discussion for further details). The disparity between plasma and CSF metabolites levels has been shown in an individual with MMDS, suggesting the manifestation of biallelic *NFU1* variants with tissue‐specific phenotypes.^25^In summary, we report 16 affected individuals with an HSP presentation of bi‐allelic *NFU1* variants and 3 affected individuals with GDD, hypotonia, longer survival, and fatal response to metabolic decompensation, which contrasts with the typical MMDS1 presentation of *NFU1* deficiency and highlights the *NFU1*‐associated disease continuum.

## Conflict of Interest

G.B. is/was a consultant for Passage Bio Inc (2020–2021) and Ionis (2019). She is/was a site investigator for the Alexander's disease trial of Ionis (2021), Metachromatic leukodystrophy of Shire/Takeda (2020–2021), Krabbe and GM1 gene therapy trials of Passage Bio, and Adrenoleukodystrophy/Hematopoietic stem cell transplantation natural history study of Bluebird Bio (2019) and a site sub‐investigator for the MPS II gene therapy trial of Regenxbio (2021). She has received an unrestricted educational grant from Takeda (2021–2022). She serves on the scientific advisory board of the Pelizaeus‐Merzbacher Foundation and is the Chair of the Medical and Scientific Advisory Board of the United Leukodystrophy Foundation. She is on the editorial boards of Neurology Genetics, Frontiers in Neurology – Neurogenetics, and Journal of Medical Genetics. RP, KA, HZE, AL and RB are employees of GeneDx, LLC.

## Supporting information


**Data S1**: Supplemental information including case reports, supplemental methods, supplemental experimental results and discussion

**Table S1**: Supplemental clinical‐genetic table
